# HIV Is Associated with Modified Humoral Immune Responses in the Setting of HIV/TB Coinfection

**DOI:** 10.1128/mSphere.00104-20

**Published:** 2020-05-20

**Authors:** Esther van Woudenbergh, Edward B. Irvine, Leela Davies, Marwou de Kock, Willem A. Hanekom, Cheryl L. Day, Sarah Fortune, Galit Alter

**Affiliations:** a Ragon Institute of MGH, MIT, and Harvard, Cambridge, Massachusetts, USA; b Department of Immunology and Infectious Diseases, Harvard T. H. Chan School of Public Health, Boston, Massachusetts, USA; c Division of Infectious Diseases, Brigham and Women’s Hospital, Boston, Massachusetts, USA; d South African Tuberculosis Vaccine Initiative (SATVI), School of Child and Adolescent Health, Institute of Infectious Diseases and Molecular Medicine, University of Cape Town, Observatory, South Africa; e Department of Microbiology and Immunology, Emory University School of Medicine, Emory University, Atlanta, Georgia, USA; University of Michigan-Ann Arbor

**Keywords:** HIV, antibodies, coinfection, humoral immunity, tuberculosis

## Abstract

Tuberculosis (TB) represents the largest cause of death in human immunodeficiency virus (HIV)-infected individuals in part due to HIV-related CD4^+^ T cell loss, rendering patients immunocompromised and susceptible to a loss of Mycobacterium tuberculosis control. However, in light of increasing data pointing to a role for humoral immunity in controlling M. tuberculosis infection, here, we aimed to define whether HIV infection also alters the humoral immune response in subjects with active and latent TB. We show that in the setting of active TB, HIV-positive individuals have significantly lower IgG responses to LAM and Ag85 than HIV-negative individuals. Furthermore, significant isotype/subclass-specific differences were frequently observed, with active TB, HIV-positive individuals demonstrating compromised antigen-specific IgM titers. HIV-infected individuals with active TB also exhibited a significant loss of influenza hemagglutinin- and tetanus toxoid-specific antibody titers at the isotype/subclass level, a symptom of broad humoral immune dysfunction likely precipitated by HIV infection. Finally, we illustrated that despite the influence of HIV infection, differences in M. tuberculosis-specific antibody profiles persist between latent and active TB disease. Taken together, these findings reveal significant HIV-associated disruptions of the humoral immune response in HIV/TB-coinfected individuals.

**IMPORTANCE** TB is the leading cause of death from a single infectious agent globally, followed by HIV. Furthermore, TB represents the leading cause of death among people with HIV. HIV is known to cause severe defects in T cell immunity, rendering HIV/TB-coinfected individuals more susceptible to TB disease progression and complicating accurate TB disease diagnosis. Here, we demonstrate that HIV infection is additionally associated with severely compromised antibody responses, particularly in individuals with active TB. Moreover, despite the influence of HIV infection, antibody profiles still allow accurate classification of individuals with active versus latent TB. These findings reveal novel immunologic challenges associated with HIV/TB coinfection and additionally provide a basis with which to leverage the key antibody features identified to potentially combat TB globally via next-generation therapeutic or diagnostic design.

## Abstract (precis)

TB is the leading cause of death from a single infectious agent globally, followed by HIV. Furthermore, TB represents the leading cause of death among people with HIV. HIV is known to cause severe defects in T cell immunity, rendering HIV/TB-coinfected individuals more susceptible to TB disease progression and complicating accurate TB disease diagnosis. Here, we demonstrate that HIV infection is additionally associated with severely compromised antibody responses, particularly in individuals with active TB. Moreover, despite the influence of HIV infection, antibody profiles still allow accurate classification of individuals with active versus latent TB. These findings reveal novel immunologic challenges associated with HIV/TB coinfection and additionally provide a basis with which to leverage the key antibody features identified to potentially combat TB globally via next-generation therapeutic or diagnostic design.

## INTRODUCTION

Tuberculosis (TB) is the leading cause of death from a single infectious agent (1), followed by human immunodeficiency virus (HIV) (2). Approximately one quarter of the world population is clinically defined as having latent TB (LTBI) (1); however, only a fraction of these individuals—five to fifteen percent—will develop active TB (ATB) disease during their lifetime (1). Left untreated, ATB is associated with 45% mortality in HIV-negative individuals (3). Conversely, HIV-positive individuals are 20 to 30 times more likely to develop ATB than HIV-negative individuals, and without treatment, the mortality of ATB in HIV-positive individuals is close to 100% (3). Ultimately, TB represents the leading cause of death among people with HIV and is responsible for approximately one-third of HIV-associated deaths globally (2). However, the precise changes in the M. tuberculosis-specific immune response that contribute to disease progression in HIV-infected individuals are incompletely understood.

HIV is known to cause immune dysfunction, rendering HIV/TB-coinfected individuals more susceptible to progression to ATB (4–6). Specifically, progressive untreated HIV infection is associated with a loss of total (4) and M. tuberculosis-specific CD4^+^ T cells (7). Given the critical role of CD4^+^ T cells in the control of TB in mice (8–10), depletion of T cells is likely to contribute to the development of ATB in HIV-infected individuals. However, antiretroviral therapy (ART)-treated and virally suppressed HIV-infected individuals with healthy CD4^+^ T cell counts (over 700 cells/mm^3^) still maintain a 4.4-fold higher rate of progression to ATB than HIV-negative individuals from the same community (11). Furthermore, even during the first year of HIV infection, when CD4^+^ T cell counts remain high, the risk of developing ATB is significantly higher in HIV-positive than HIV-negative individuals (12, 13). These data suggest that HIV disrupts additional immunologic drivers of TB control, beyond CD4^+^ T cell immunity, that are yet to be defined.

In this respect, HIV infection additionally results in significant aberrations in the B cell compartment and thus humoral immunity (14). For example, HIV infection is associated with hypergammaglobulinemia (15–18), likely due to HIV-induced B cell hyperactivity. HIV-infected individuals additionally display lower levels of memory B cells (19–21) and impaired antibody responses to pneumococcus, tetanus toxoid, and influenza (22, 23). Studies into the humoral response during HIV/TB coinfection are limited; however, a few studies have reported reduced IgG responses in individuals with HIV/TB coinfection (24–28). For example, HIV/TB-coinfected individuals were observed to have lower arabinomannan (AM)-specific total IgG titers (26) and lower lipoarabinomannan (LAM)-specific IgG subclass titers (24, 27) than HIV-uninfected individuals. Along these same lines, serum IgG levels against M. tuberculosis purified protein derivative (PPD) have been shown to decline with HIV disease progression (28). Collectively, these data suggest that HIV/TB-coinfected individuals display lower M. tuberculosis-specific IgG titers.

However, whether similar changes are observed across distinct antibody isotypes is poorly defined. Furthermore, studies to date have largely been restricted to the analysis of antibody titers to a few antigens, with limited characterization of antibody levels to additional M. tuberculosis antigens, of antibody levels to control antigens, or of antibody Fc functionality in these populations. Thus, given the increasing evidence pointing to a protective role for antibodies during M. tuberculosis infection (29–36), here, we performed a comprehensive, agnostic characterization of antibody profiles across multiple antigens in HIV-infected and -uninfected ATB and LTBI individuals from Cape Town, South Africa (Table 1), with the goal of identifying HIV-associated disruptions of humoral immunity that may result in reduced immune pressure on M. tuberculosis.

**TABLE 1:** Cohort demographic data and HIV-associated treatment and immune parameters

Parameter	Value by group				
	ATB/HIV+ (*n* = 15)	ATB/HIV− (*n* = 28)	LTBI/HIV+ (*n* = 24)	LTBI/HIV− (*n* = 25)	Control (*n* = 8)
Demographics					
Mean age (years ± SD)	35.5 ± 6.96	40.6 ± 9.70	33.1 ± 7.57	28.6 ± 8.26	38.4 ± 15.1
Female (*n* [%])	8/15 (53)	11/28 (39)	19/24 (79)	9/25 (36)	2/8 (25)
HIV parameters					
ART treatment	6/15 (40)		0/24 (0)		
CD4+ T cell count mean (cells/mm^3^ ± SD)	189.3 ± 164.9		485.3 ± 291.4		
Viral load mean (copies/ml ± SD)	185,353 ± 322,623		37,977 ± 60,557		

## RESULTS

### Bulk immunoglobulin levels are increased during HIV and TB infection.

Hypergammaglobulinemia, a hallmark of humoral immune dysfunction as a consequence of chronic immune activation, has been noted in both HIV and TB infections (18, 37). However, whether these changes are amplified or altered across antibody isotypes and subclasses in the setting of HIV/TB coinfection is largely unknown. Thus, we first determined the overall levels of bulk IgG1, IgG2, IgG3, IgG4, IgA, and IgM in the plasma of actively (ATB) and latently (LTBI) TB-infected individuals with and without HIV coinfection, as well as in the plasma of negative-control individuals. Consistent with previous work (18, 37), hypergammaglobulinemia in both HIV- and M. tuberculosis-infected individuals was observed.

**FIG 1:**
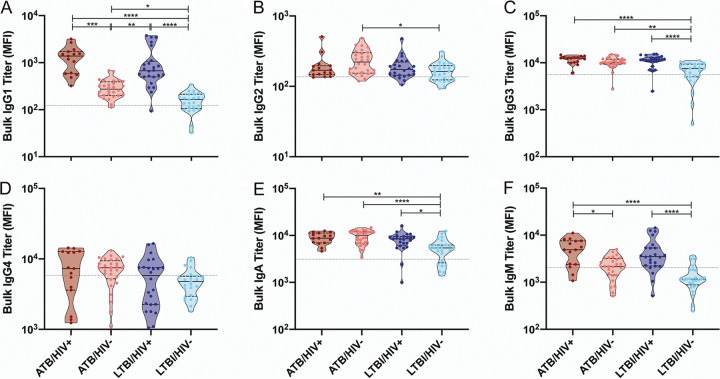
Bulk immunoglobulin levels are increased during HIV and M. tuberculosis infection. Relative levels of bulk IgG1 (A), IgG2 (B), IgG3 (C), IgG4 (D), IgA (E), and IgM (F) present in the plasma of the study population and negative controls were determined via Luminex. The median fluorescence intensity (MFI) for each individual is graphed. The gray dotted lines is the median of the control group. Within each violin plot, the black solid line is the median and the black dashed lines show the interquartile range. Kruskal-Wallis with Dunn’s multiple-comparison test was used. Adjusted *P* values are as follows: *, *P* < 0.05; **, *P* < 0.01; ***, *P* < 0.001; ****, *P* < 0.0001.

Specifically, a significant increase in the abundance of most subclasses, excluding IgG4 and IgM, was observed in the plasma of the ATB/HIV− group compared with the LTBI/HIV− group, highlighting hypergammaglobulinemia attributable to active TB disease (Fig. 1A to F). Moreover, bulk IgG1 and IgM levels were significantly higher in HIV/TB-coinfected individuals compared with individuals with ATB or LTBI alone (Fig. 1A and F), and IgG3 and IgA levels were significantly higher in LTBI/HIV+ individuals than in LTBI/HIV− individuals (Fig. 1C and E). These differences suggest that even in the setting of ATB and LTBI, HIV infection further exacerbates hypergammaglobulinemia. Finally, no significant differences in bulk antibody levels were detected between the ATB/HIV+ and LTBI/HIV+ groups (Fig. 1A to F), indicating that more severe TB disease does not further intensify hypergammaglobulinemia in HIV-infected individuals.

Together, these data imply that ATB, and to a greater extent HIV, induce elevated bulk immunoglobulin levels in the plasma of afflicted individuals.

### HIV infection is associated with modified M. tuberculosis-specific total IgG titers.

To determine whether the elevated bulk IgG levels observed were linked to elevated M. tuberculosis-specific IgG titers, total IgG levels were assessed across a set of four M. tuberculosis antigens, namely, purified protein derivative (PPD), lipoarabinomannan (LAM), Ag85A/B, and ESAT6/CFP10. PPD is a heterogenous compilation of M. tuberculosis proteins (38), LAM is a critical cell wall glycolipid (39), Ag85 is a secreted virulence factor linked to cell wall biosynthesis and host cell invasion (40–42), and ESAT6 and CFP10 represent secreted virulence factors involved in phagosomal rupture (43). In addition, total IgG levels were measured for HIV gp120, as well as for three control antigens, namely, influenza hemagglutinin (HA), tetanus toxoid, and pneumococcal polysaccharide (PPSV23) (44).

**FIG 2:**
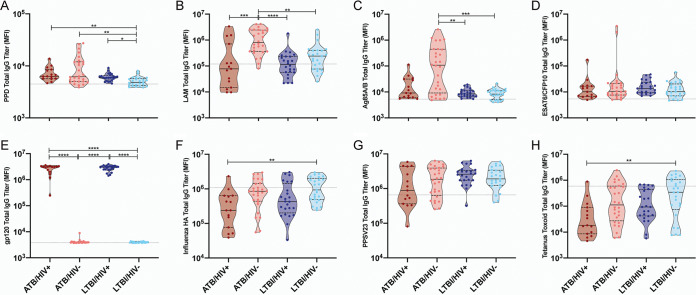
HIV infection is associated with modified M. tuberculosis-specific total IgG titers. Relative levels of IgG specific to PPD (A), LAM (B), Ag85A/B (C), ESAT6/CFP10 (D), HIV gp120 (E), influenza HA (F), PPSV23 (G), and tetanus toxoid (H) present in the plasma of the study population and negative controls were determined via Luminex. The MFI for each individual is graphed. The gray dotted line is the median of the control group. Within each violin plot, the black solid line is the median and the black dashed lines show the interquartile range. Kruskal-Wallis with Dunn’s multiple-comparison test was used. Adjusted *P* values are as follows: *, *P* < 0.05; **, *P* < 0.01; ***, *P* < 0.001; ****, *P* < 0.0001.

First, as expected (32, 45–47), in the HIV-negative setting, ATB individuals exhibited increased total IgG titers to multiple M. tuberculosis antigens. Specifically, IgG responses to PPD, LAM, and Ag85A/B were significantly higher in ATB/HIV− individuals than in LTBI/HIV− individuals (Fig. 2A to C). Furthermore, the disease state of M. tuberculosis infection did not significantly alter total IgG titers to HIV gp120 or any of the three control antigens tested (Fig. 2E to H).

Next, we evaluated whether HIV infection was associated with significant modifications in M. tuberculosis-specific IgG titers in ATB and/or in LTBI individuals. Most strikingly, despite higher bulk, nonspecific IgG titers, ATB/HIV+ individuals exhibited significantly lower levels of LAM-specific IgG than ATB/HIV− individuals (Fig. 2B). ATB/HIV+ individuals also displayed a trend toward lower levels of Ag85-specific IgG, although this difference was not significant (Fig. 2C). In contrast, no difference in PPD or ESAT6/CFP10 IgG titer was observed when the ATB/HIV+ and ATB/HIV− groups were compared (Fig. 2A and D). Similarly, no differences were observed in influenza HA-, PPSV23-, or tetanus-specific responses across the ATB/HIV+ and ATB/HIV− subjects (Fig. 2F to H). In the context of LTBI, HIV-positive individuals had moderately, yet significantly higher levels of PPD-reactive IgG than HIV-negative individuals (Fig. 2A), whereas no significant differences were observed across additional M. tuberculosis antigens or non-M. tuberculosis antigens, with the exception of HIV gp120 (Fig. 2B to H).

Thus, these data indicate that HIV coinfection is associated with compromised LAM-specific total IgG titers during ATB, yet potentially augmented PPD-reactive humoral immunity in the setting of LTBI. Ultimately, these data point to specific alterations in the humoral immune response to M. tuberculosis with HIV infection that occur independently of hypergammaglobulinemia and that may reflect the altered biology of infection.

### ATB/HIV+ individuals exhibit a broad decline in pathogen-specific humoral immunity.

**FIG 3:**
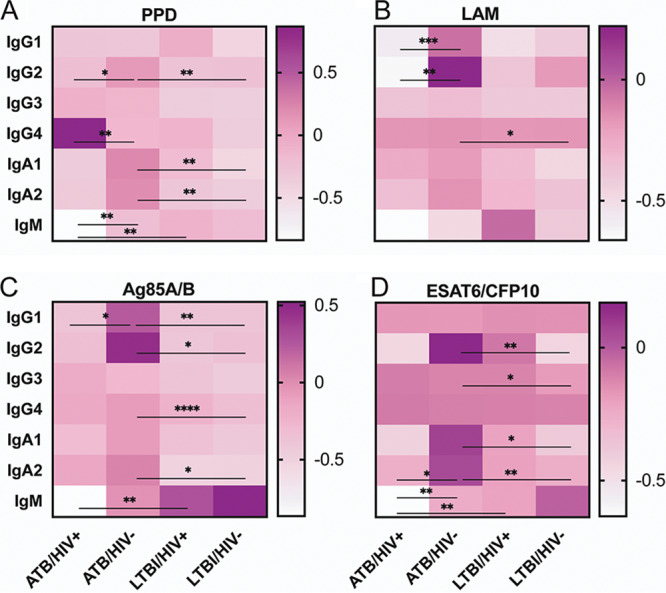
HIV infection is associated with decreased M. tuberculosis-specific isotype and subclass titers during ATB. Relative levels of the indicated antibody isotype/subclass specific to PPD (A), LAM (B), Ag85A/B (C), and ESAT6/CFP10 (D) present in the plasma of the study population were determined via Luminex. Following data collection, a Z-score transformation was applied to each antibody variable. Next, the median value of each infection group was graphed to summarize group reactivity to each antigen. Kruskal-Wallis with Dunn’s multiple-comparison test was performed on the untransformed data to assess significance. Adjusted *P* values are as follows: *, *P* < 0.05; **, *P* < 0.01; ***, *P* < 0.001; ****, *P* < 0.0001. Statistical significance comparing ATB/HIV+ and LTBI/HIV−, as well as ATB/HIV− and LTBI/HIV+, are not shown here (see Fig. S1 and S2).

Beyond changes in overall IgG titers, additional differences in isotype and IgG subclass selection have been reported during M. tuberculosis infection (48, 49). Thus, relative isotype/subclass titers to the same collection of antigens described above were next measured for each infection group. Following data collection, a Z-score transformation was applied to each antibody variable for ease of visualization. Next, the median value of each infection group was plotted in a heatmap to summarize group reactivity to each antigen (Fig. 3). The untransformed data were additionally included in Fig. S1 and S2 in the supplemental material.

10.1128/mSphere.00104-20.1 FIG S1 HIV infection is associated with modified IgG subclass titers during ATB. Relative levels of IgG1 (A), IgG2 (B), IgG3 (C), and IgG4 (D) specific to PPD, LAM, Ag85A/B, ESAT6/CFP10, HIV gp120, influenza HA, PPSV23, and tetanus toxoid present in the plasma of the study population and negative controls were determined via Luminex. MFI for each individual is graphed. The grey dotted line is the median of the control group. Within each violin plot, the black solid line is the median and the black dashed lines show the interquartile range. Kruskal-Wallis with Dunn’s multiple-comparison test was used. Adjusted *P* values are as follows: *, *P* < 0.05; **, *P* < 0.01; ***, *P* < 0.001; ****, *P* < 0.0001. Download FIG S1, PDF file, 2.2 MB. Copyright © 2020 van Woudenbergh et al. 2020 van Woudenbergh et al. This content is distributed under the terms of the Creative Commons Attribution 4.0 International license.

10.1128/mSphere.00104-20.2 FIG S2 HIV infection is associated with modified IgM and IgA titers during ATB. Relative levels of IgM (A), IgA1 (B), and IgA2 (C)specific to PPD, LAM, Ag85A/B, ESAT6/CFP10, HIV gp120, influenza HA, PPSV23, and tetanus toxoid present in the plasma of the study population and negative controls were determined via Luminex. The MFI for each individual is graphed. The grey dotted line is the median of the control group. Within each violin plot, the black solid line is the median and the black dashed lines show the interquartile range. Kruskal-Wallis with Dunn’s multiple-comparison test was used. Adjusted *P* values are as follows: *, *P* < 0.05; **, *P* < 0.01; ***, *P* < 0.001; ****, *P* < 0.0001. Download FIG S2, PDF file, 1.9 MB. Copyright © 2020 van Woudenbergh et al. 2020 van Woudenbergh et al. This content is distributed under the terms of the Creative Commons Attribution 4.0 International license.

First, in the absence of HIV infection, ATB individuals exhibited higher levels of IgG and IgA subclass titers to PPD, Ag85, and ESAT6/CFP10 than LTBI individuals (Fig. 3A, C, and D; Fig. S1 and S2), consistent with previous work describing higher M. tuberculosis-specific antibody titers in active disease (32, 45–47). Conversely, ATB individuals did not have increased M. tuberculosis-specific titers compared with LTBI individuals in the setting of HIV infection. Instead, remarkably, LTBI/HIV+ individuals had increased IgM titers to each M. tuberculosis antigen compared with ATB/HIV+ individuals, although the difference in LAM-specific IgM did not reach statistical significance (Fig. 3A to D; Fig. S2A).

We next sought to test the hypothesis that in the setting of ATB and LTBI, HIV infection modifies the selection of particular antigen-specific isotypes and subclasses. Consistent with the trend of decreased total IgG titer to LAM and Ag85 observed in ATB/HIV+ individuals, this group additionally exhibited significantly lower IgG1 titers to both of these antigens than the ATB/HIV− group (Fig. 3B and C; Fig. S1A). Furthermore, PPD- and LAM-specific IgG2 levels were significantly lower in ATB/HIV+ individuals than in ATB/HIV− individuals (Fig. 3A and B; Fig. S1B). PPD- and ESAT6/CFP10-specific IgM levels were additionally significantly lower in ATB/HIV+ individuals than in ATB/HIV− individuals (Fig. 3A and D; Fig. S2A), despite elevated levels of bulk IgM in the overall plasma pool (Fig. 1F). Indeed, lower IgG2 and lower IgM titers in ATB/HIV+ than in ATB/HIV− were clear trends observed across all M. tuberculosis antigens tested (Fig. 3A to D), albeit the magnitude of difference in some cases was small (Fig. S1B and S2A). Conversely, PPD-specific IgG4 titers were the only antibody isotype/subclass titer that was significantly higher in the ATB/HIV+ group than in the ATB/HIV− group (Fig. 3A; Fig. S1D). Finally, no significant differences were observed in isotype/subclass levels to any of the M. tuberculosis antigens tested in the LTBI/HIV+ group compared with the LTBI/HIV− group (Fig. 3A to D; Fig. S1 and S2).

Isotype and subclass responses to influenza HA, tetanus toxoid, and PPSV23 were additionally measured to determine if HIV-associated alterations in antibody isotype/subclass levels observed were unique to M. tuberculosis antigens. In this respect, HIV/TB-coinfection did not significantly alter isotype/subclass titers to PPSV23 (Fig. S1 and S2). However, ATB/HIV+ individuals did exhibit significantly reduced IgG2, IgG4, IgA1, IgA2, and IgM titers to tetanus toxoid (Fig. S1 and S2), as well as reduced IgG1, IgG2, and IgM titers to influenza HA compared with ATB/HIV− individuals (Fig. S1 and S2).

Taken together, these data reveal that ATB/HIV+ individuals exhibit decreased M. tuberculosis-specific antibody titers across multiple antibody isotypes and subclasses. These reduced titers are additionally observed to influenza HA and tetanus toxoid and are thus likely a symptom of a broader decline of pathogen-specific humoral immunity precipitated by HIV infection.

### Antigen-specific IgM and IgG4 titers correlate with CD4^+^ T cell counts.

Given the striking differences in antigen-specific humoral immunity observed within and between infection groups, we next sought to determine whether HIV-associated treatment or immune status (Table 1) might impact differences in M. tuberculosis-specific humoral immunity.

**FIG 4:**
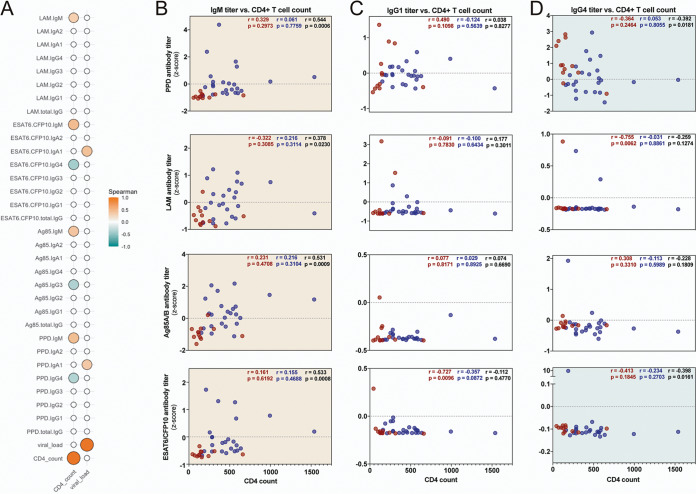
Antigen-specific IgM and IgG4 titers correlate with CD4^+^ T cell counts. (A) Spearman correlations across all HIV+ individuals comparing CD4^+^ T cell counts and viral loads to Z-scored antigen-specific isotype and subclass titers. Statistically significant correlations (*P* < 0.05) are indicated by the filled circles. The size and color of each correlation circle corresponds to the strength of the relationship. Correlations with *P* value of >0.05 are empty, white circles. (B to D) Correlation plots of selected IgM (B), IgG1 (C), and IgG4 (D) data from panel A. Each dot represents an individual, with ATB/HIV+ in red and LTBI/HIV+ in blue. In the top right of each graph, Spearman *r* and *P* values are indicated for ATB/HIV+ (red), LTBI/HIV+ (blue), and total HIV+ (black). The background of each graph is colored to indicate the significance and directionality of the correlation across all HIV+ individuals. Orange, significant positive correlation; teal, significant negative correlation; white, nonsignificant correlation.

A critical function of CD4^+^ T cells is to augment humoral immunity by driving B cell survival and proliferation, antibody affinity maturation, and antibody class switching, as well as the development of long-lived antibody secreting plasma cells and memory B cells (50–52). Given the loss of CD4^+^ T cells with progressive HIV infection (53), we first examined whether changes in M. tuberculosis-specific humoral immunity in HIV-infected individuals were linked to changes in CD4^+^ T cell counts. Spearman correlations highlighted significant relationships between M. tuberculosis-specific IgM titers and CD4^+^ T cell counts across the whole cohort of HIV-infected individuals (Fig. 4A and B). However, these correlations were lost when ATB/HIV+ and LTBI/HIV+ individuals were analyzed separately, as demonstrated in the univariate correlation plots (Fig. 4B).

With respect to IgG, while a number of M. tuberculosis-specific total IgG, IgG1, and IgG2 features were lower in ATB/HIV+ individuals than in ATB/HIV− individuals (Fig. 2 and 3), no significant positive correlations were observed with CD4^+^ T cell counts (Fig. 4A and C), suggesting that weaker M. tuberculosis-specific IgG responses may not strictly be attributable to lower CD4^+^ T cell counts. Conversely, PPD and ESAT6/CFP10 IgG4 levels were found to have a significant negative correlation with CD4^+^ T cell counts, and a similar trend was observed for LAM- and Ag85A/B-specific IgG4 levels (Fig. 4A and D). Given the relatively low affinity of IgG4 for activating Fcγ receptors (FcγRs) and its role in reducing polyclonal antibody effector function (54, 55), these data may point to the importance of CD4^+^ T cell numbers in maintaining effective humoral subclass selection profiles in the setting of HIV/TB coinfection.

To further probe the direct relationships between HIV loads and M. tuberculosis-specific antibody levels, correlations between viral load and each M. tuberculosis-specific antibody titer were performed. Only two significant correlations were observed, as PPD IgA1 and ESAT6/CFP10 IgA1 levels were each found to have a significant, positive correlation with viral load (Fig. 4A).

Finally, 6 of 15 ATB/HIV+ individuals reported that they were on antiretroviral therapy (ART) (Table 1). Thus, we additionally probed antibody profiles in the ATB/HIV+ group in a manner stratified by ART status. As expected, HIV loads were significantly lower among subjects on ART (see Table S1 in the supplemental material). However, no significant differences were observed across any of the M. tuberculosis-specific antibody titer measurements (Table S1). Therefore, differences in M. tuberculosis-specific antibody levels are unlikely strictly related to the use of ART. However, this analysis is limited by a small sample size and the unclear reliability of self-reported ART status.

10.1128/mSphere.00104-20.5 TABLE S1 No significant difference in M. tuberculosis-specific antibody levels related to ART status. ATB/HIV+ individuals were stratified by ART status. Next, a Mann-Whitney U test was applied to viral load, CD4+ T cell count, and each M. tuberculosis-specific antibody feature individually. *P* values and *q*-values from each test are indicated. The *q*-values were calculated using the Benjamini-Hochberg procedure (77). Download Table S1, PDF file, 0.05 MB. Copyright © 2020 van Woudenbergh et al. 2020 van Woudenbergh et al. This content is distributed under the terms of the Creative Commons Attribution 4.0 International license.

Taken together, these data suggest that a subset of differences in M. tuberculosis-specific antibody levels—most intriguingly IgM and IgG4 titers—may be linked to CD4^+^ T cell and HIV infection dynamics. Conversely, there was no evidence to suggest that M. tuberculosis-specific antibody titer differences were strictly related to the use of ART.

### HIV-infected individuals exhibit an M. tuberculosis-specific antibody profile defined by compromised LAM and Ag85 responses and reduced humoral immune coordination.

To approach a more holistic understanding of the coordinated aspects of the M. tuberculosis-specific humoral immune response disrupted by HIV infection beyond differences in antibody titer, the ability of M. tuberculosis-specific antibodies to bind FcγRs was captured (see Fig. S3 in the supplemental material), as was the ability of PPD-specific antibodies to drive antibody-dependent cellular phagocytosis (ADCP), antibody-dependent neutrophil phagocytosis (ADNP), and antibody dependent NK cell activation (ADNKA) (see Fig. S4 in the supplemental material). This resulted in the collection of 49 unique M. tuberculosis-specific antibody Fc features for each individual plasma sample.

10.1128/mSphere.00104-20.3 FIG S3 HIV infection is associated with decreased LAM- and Ag85-specific Fcγ receptor binding activity during ATB. Relative FcγRIIa (A), FcγRIIb (B), and FcγRIIIa (C) binding activity specific to PPD, LAM, Ag85A/B, and ESAT/CFP10 (left to right) present in the plasma of the study population and negative controls were determined via Luminex. The MFI for each individual is graphed. The grey dotted line is the median of the control group. Within each violin plot, the black solid line is the median and the black dashed lines show the interquartile range. Kruskal-Wallis with Dunn’s multiple-comparison test was used. Adjusted *P* values are as follows: *, *P* < 0.05; **, *P* < 0.01; ***, *P* < 0.001; ****, *P* < 0.0001. Download FIG S3, PDF file, 0.9 MB. Copyright © 2020 van Woudenbergh et al. 2020 van Woudenbergh et al. This content is distributed under the terms of the Creative Commons Attribution 4.0 International license.

10.1128/mSphere.00104-20.4 FIG S4 PPD-specific antibodies from ATB individuals drive increased innate immune activation in the setting of HIV infection. PPD-specific antibodies in the plasma from each individual was tested for their ability to drive Fc-mediated effector functions in innate immune cells. (A to C) Antibody-dependent NK cell activation by primary human NK cells. (D) Antibody-dependent cellular phagocytosis by THP-1 monocytes. (E) Antibody-dependent neutrophil phagocytosis by primary human neutrophils. For each graph, the grey dotted line is the median of the control group. Within each violin plot, the black solid line is the median and the black dashed lines show the interquartile range. Kruskal-Wallis with Dunn’s multiple-comparison test was used. Adjusted *P* values are as follows: *, *P* < 0.05; **, *P* < 0.01; ***, *P* < 0.001; ****, *P* < 0.0001. Download FIG S4, PDF file, 1.5 MB. Copyright © 2020 van Woudenbergh et al. 2020 van Woudenbergh et al. This content is distributed under the terms of the Creative Commons Attribution 4.0 International license.

We next aimed to use the full M. tuberculosis-specific antibody profile amassed for determining the specific impact of HIV infection on the M. tuberculosis-specific humoral immune response in the setting of ATB and LTBI by taking a computational approach. First, data from ATB and LTBI subjects were segregated. Then, to mitigate data overfitting and augment model interpretability, least absolute shrinkage and selection operator (LASSO) feature selection was performed. Next, partial least-squares discriminant analysis (PLS-DA) classification models were generated using the LASSO-selected features, and the latent variables from the PLS-DA model were graphed to visualize the extent of group separation (56, 57). Five-fold repeated cross-validation (CV) was performed to assess the accuracy of each model; furthermore, the performance of each model was compared with the performance of a permuted model to assess model significance.

**FIG 5:**
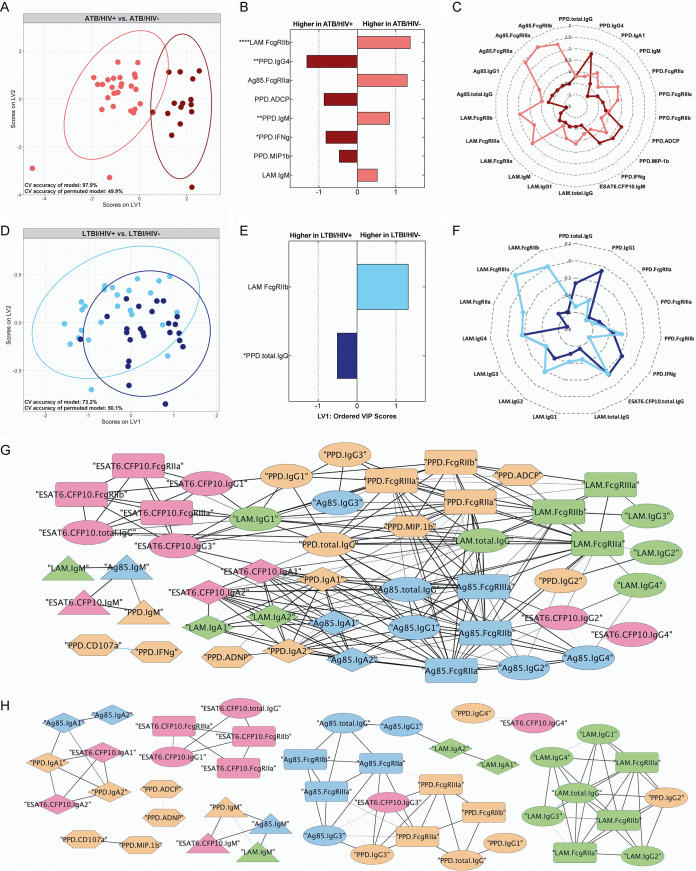
HIV-positive and HIV-negative M. tuberculosis-infected individuals exhibit divergent M. tuberculosis-specific humoral profiles. Multivariate analyses comparing M. tuberculosis-specific antibody responses elicited by HIV-positive and HIV-negative individuals. (A and D) Visualization from PLS-DA models trained using the LASSO-selected features in the ATB (A) and LTBI (D) subset of individuals. Ellipses represent 95% confidence intervals. Cross validation (CV) accuracy of the models and permuted models are indicated. The accuracy of each model is significantly higher than that of the respective permuted model (Mann-Whitney U test ATB model, *P* < 2.2e^−16^; Mann-Whitney U test LTBI model, *P* < 2.2e^−16^). (B and E) LASSO-selected features from the ATB (B) and LTBI models (E) are plotted on variable importance in the projection (VIP) plots. Variables with VIP scores greater than 1 contribute most to separation across latent variable 1 (LV1). LASSO-selected features significantly different across the groups during univariate analyses are indicated. Kruskal-Wallis with Dunn’s multiple-comparison test was used. Adjusted *P* values are as follows: *, *P* < 0.05; **, *P* < 0.01; ***, *P* < 0.001; ****, *P* < 0.0001. (C and F) Radar plots of each LASSO-selected feature and its significant correlates in the ATB (C) and LTBI (F) models. Median Z-score of each group is plotted for a given feature. (G and H) Correlation networks depict significant correlations within the M. tuberculosis-specific antibody response in HIV-negative (G) and HIV-positive (H) individuals. LTBI and ATB individuals were combined to make each correlation network. The width of each edge corresponds to the magnitude of the Spearman *r* values. Colors: orange (PPD), green (LAM), blue (Ag85A/B), pink (ESAT6/CFP10). Shapes: ellipse (IgG titer), diamond (IgA titer), triangle (IgM titer), rounded rectangle (Fc-receptor binding), hexagon (functional assay). Significant correlations were defined as those with *q* value of <0.01. The *q* values were calculated using the Benjamini-Hochberg procedure (77).

In the setting of ATB, robust separation in the M. tuberculosis-specific humoral immune profile was observed between HIV-positive and -negative individuals, even excluding the HIV gp120 data collected (Fig. 5A). Specifically, the PLS-DA model generated could distinguish ATB/HIV+ from ATB/HIV− individuals with 97.9% accuracy (Fig. 5A). Two of the top discriminatory features identified as lower in ATB/HIV+ individuals than that in ATB/HIV− individuals included LAM-specific antibody binding to FcγRIIb and Ag85-specific antibody binding to FcγRIIa (Fig. 5B). PPD- and LAM-specific IgM additionally contributed to class separation, albeit to a lesser extent (Fig. 5B). Conversely, as observed in the univariate analysis, PPD-specific IgG4 titers were elevated in ATB/HIV+ individuals. ATB/HIV+ individuals additionally possessed higher levels of PPD-specific phagocytic (ADCP) and NK cell (interferon gamma [IFN-γ] and MIP1β) activating antibodies (Fig. 5B). Finally, examination of the significant correlates of each of these discriminatory antibody features revealed that the ATB/HIV+ group demonstrated widely compromised LAM- and Ag85-specific antibody responses compared with the ATB/HIV− group, as illustrated by the differences in the group median for each of these features on the radar plot (Fig. 5C).

HIV coinfection additionally altered M. tuberculosis-specific humoral immunity in latent infection, although to a lesser extent (Fig. 5D). More specifically, the PLS-DA model generated, excluding the HIV gp120 data collected, could distinguish LTBI/HIV+ from LTBI/HIV− individuals with 73.2% accuracy (Fig. 5D). Furthermore, the model found that LAM-specific FcγRIIb binding was selectively enriched among LTBI/HIV− individuals, whereas PPD-specific IgG titers were enhanced in LTBI/HIV+ individuals (Fig. 5E). Further analysis of the significant correlates of these two discriminatory features highlights a pattern consistent with that observed from the ATB model. Specifically, numerous features of the LAM-specific antibody response—most notably LAM FcγR binding—were higher in the HIV-negative group, reflecting a more functional LAM response in LTBI/HIV− individuals (Fig. 5F). In contrast, a trend toward stronger PPD responses was observed in LTBI/HIV+ individuals (Fig. 5F).

Given that humoral features are all tightly coregulated, we next aimed to define whether distinct relationships existed among the features in the presence/absence of HIV infection. Thus, using the 49 M. tuberculosis-specific antibody features captured, individual correlation networks were generated for HIV-negative and HIV-positive individuals. In contrast to the analyses above, here, the LTBI and ATB groups were combined to increase the dynamic range of the data for more robust correlation analyses. A dense network of significantly correlated relationships were observed in HIV-negative individuals, highlighting the highly coordinated nature of the M. tuberculosis-specific humoral immune response in this infection setting (Fig. 5G). In contrast, the correlation network was less dense and connected in the HIV-positive setting, largely restricted to a single antigen or antibody isotype (Fig. 5H). For example, in HIV-positive group, ESAT6/CFP10 responses strongly correlated within that antigen target but correlated poorly across additional specificities, including PPD, LAM, and Ag85 responses (Fig. 5H). Similarly, the IgA response correlated across multiple antigens in the HIV-positive group; however, in contrast to the HIV-negative correlation network, these responses were not linked to additional features of the humoral immune response (Fig. 5H).

Collectively, these data imply that HIV infection drives considerable differences in M. tuberculosis-specific humoral immunity, both at a univariate level and at the level of humoral immune coordination. M. tuberculosis-specific antibody features distinguish HIV-positive from HIV-negative individuals in the setting of ATB and LTBI, with HIV-positive individuals displaying compromised LAM- and Ag85-targeting antibody responses, yet slightly augmented PPD-specific titers and antibody effector functions. Furthermore, beyond these differences in individual humoral parameters, correlation analyses suggest that HIV infection disrupts the coordination of the M. tuberculosis-specific humoral immune response in coinfected individuals.

### Humoral immune profiles distinguish ATB from LTBI irrespective of HIV status.

Recent data illustrate that ATB and LTBI individuals display distinct antibody profiles (32, 58); however, whether these profiles remain distinct in the setting of HIV coinfection, particularly given the profound changes in the M. tuberculosis-specific humoral response associated with HIV infection, is unclear. Thus, to test the hypothesis that the discriminatory power of antibodies in separating ATB and LTBI individuals is maintained even in the setting of HIV infection, a computational approach combining LASSO feature selection and PLS-DA classification was taken as described above. However, here, data from HIV-negative and HIV-positive subjects were segregated prior to supervised analyses.

**FIG 6:**
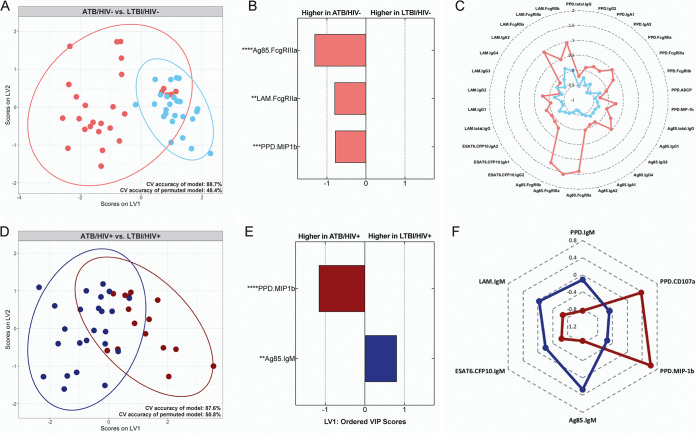
M. tuberculosis-specific antibody profiles distinguish LTBI from ATB irrespective of HIV status. Supervised analyses comparing M. tuberculosis-specific antibody responses elicited by ATB and LTBI individuals. (A and D) Visualization from PLS-DA models trained using the LASSO-selected features in the HIV-negative (A) and HIV-positive (B) subset of individuals. Ellipses represent 95% confidence intervals. CV accuracy of the models and permuted models are indicated. The accuracy of each model is significantly higher than that of the respective permuted model (Mann-Whitney U test HIV negative model, *P* < 2.2e^−16^; Mann-Whitney U test HIV positive model, *P* < 2.2e^−16^). (B and E) LASSO-selected features from the HIV-negative (B) and HIV-positive (E) models are plotted on VIP plots. Variables with VIP scores greater than 1 contribute most to separation across LV1. LASSO-selected features significantly different across the groups during univariate analyses are indicated. Kruskal-Wallis with Dunn’s multiple-comparison test was used. Adjusted *P* values are as follows: *, *P* < 0.05; **, *P* < 0.01; ***, *P* < 0.001; ****, *P* < 0.0001. (C and F) Radar plots of each LASSO-selected feature and its significant correlates in the HIV-negative (C) and HIV-positive (F) models. Median Z-score of each group is plotted for a given feature. Significant correlations were defined as those with *q* values of <0.01. The *q* values were calculated using the Benjamini-Hochberg procedure (77).

As expected, M. tuberculosis-specific antibody Fc profiles were largely able to separate ATB/HIV− from LTBI/HIV− individuals, as the model had a CV accuracy of 88.7% (Fig. 6A). Each of the features required to separate the two groups were enriched in the ATB/HIV− group, including increased levels of FcγR binding antibodies to Ag85 and LAM and PPD-specific antibodies able to drive NK cell activation (PPD.MIP1β) (Fig. 6B). An analysis of the direct correlates of each discriminatory variable indicates that a myriad of antibody features are significantly correlated with those selected by the model and are each higher in the ATB/HIV− group (Fig. 6C). This result is symptomatic of broadly increased M. tuberculosis-specific antibody titers in ATB compared with LTBI individuals.

Remarkably, in the setting of HIV infection, ATB and LTBI individuals could still be distinguished by their M. tuberculosis-specific antibody profiles, despite the severe humoral immune perturbations associated with HIV infection. Specifically, the model could distinguish ATB/HIV+ from LTBI/HIV+ individuals with 87.6% accuracy (Fig. 6D)—an accuracy nearly equivalent to that of the HIV-negative model (Fig. 6A). Only 2 of the 49 features were required to separate ATB/HIV+ from LTBI/HIV+ subjects, namely, elevated levels of PPD-specific NK cell-activating (MIP1β) antibodies in ATB/HIV+ individuals and elevated Ag85-specific IgM in LTBI/HIV+ individuals (Fig. 6E). MIP1β significantly correlated with CD107a expression and was higher in the ATB/HIV+ group (Fig. 6F). While overall differences between groups are modest in magnitude (Fig. S4), these data reflect an ability of PPD-reactive antibodies from ATB/HIV+ individuals to drive increased ADNKA *in vitro* compared with PPD-reactive antibodies from LTBI/HIV+ individuals (Fig. 6F). Conversely, IgM titers to PPD, LAM, and ESAT6/CFP10 were each significant correlates of Ag85 IgM and higher in the LTBI/HIV+ group (Fig. 6F).

Together, these data demonstrate that M. tuberculosis-specific antibody profiles can distinguish ATB from LTBI, even in the setting of HIV infection, a state in which M. tuberculosis-specific humoral immunity is disrupted. While differences between ATB and LTBI in the HIV-negative setting are broad and titer driven, in the setting of HIV infection, ATB individuals specifically exhibit a compromised M. tuberculosis-specific IgM response, allowing separation of the two populations.

## DISCUSSION

The collision of the HIV and M. tuberculosis epidemics represents one of the most devastating disease intersections globally. HIV infection results in 20 to 30 times higher risk of developing active disease among those infected with M. tuberculosis (4–6), which is partly attributable to a loss of CD4^+^ T cells with HIV disease progression (4). Yet, TB progression in HIV-infected individuals occurs at a higher rate even among those with healthy CD4^+^ T cell counts (11–13), suggesting that other immune factors may contribute to the loss of M. tuberculosis control. Given the emerging role for antibodies in M. tuberculosis control (31–36, 59, 60), we hypothesized that HIV coinfection may alter the humoral immune response to M. tuberculosis. Indeed, the data presented herein highlight the significant impact of HIV on M. tuberculosis-specific humoral immunity, irrespective of HIV-associated hypergammaglobulinemia. Changes in M. tuberculosis-specific antibody titers differed across antigens and isotype/subclass, with more profound alterations observed in ATB individuals. Yet, despite the HIV-associated changes in the humoral immune response, differences in humoral immunity across ATB and LTBI continue to discriminate the populations, highlighting the presence of some common humoral changes in HIV-positive and -negative individuals that track with a differential control of M. tuberculosis.

Among the most intriguing alterations with HIV infection was the loss of LAM- and Ag85-specific IgG responses (Fig. 2 and 3; Fig. S1), because increasing data point to a role for LAM- and Ag85-specific IgG responses in the improved control of TB disease. Specifically, passive transfer of a LAM-specific monoclonal antibody was found to provide a significant, dose-dependent reduction in M. tuberculosis burden in the lung and spleen of infected mice, as well as to significantly prolong survival (33, 34). Furthermore, a study in children found that LAM-specific IgG titers correlated with a decreased risk of disseminated TB disease, suggesting that compromised LAM-specific humoral immunity increased the likelihood of disseminated disease (61). Similarly, Ag85 IgG responses have been reported to be associated with improved TB disease outcome in a cohort of pulmonary tuberculosis patients (62). Consistent with this notion, although the MV85A phase 2b TB vaccine trial (63)—the first large TB vaccine clinical trial since BCG—was met with little clinical success, a *post hoc* correlate analysis identified Ag85A-specific IgG to be associated with a reduced risk of TB (64), pointing to Ag85 IgG as an unpredicted correlate of protection in this study. Here, we observed a significant loss of LAM- and Ag85-specific IgG responses in ATB/HIV+ individuals (Fig. 2 and 3; Fig. S1), moreover, we also observed a trend toward lower LAM-specific responses in LTBI/HIV+ individuals (Fig. 5; Fig. S3). Whether the loss of these specific antibody populations results in a loss of bacterial control remains unclear, but given the associations of LAM- and Ag85-targeting IgG with improved TB outcome, it is possible that this humoral immune deficiency could contribute to a loss of immune protection.

The loss in antigen-specific antibody titers during HIV coinfection was not exclusive to LAM- and Ag85-specific humoral immunity, as revealed by profiling at the isotype/subclass level. ATB/HIV+ subjects exhibited reduced ESAT6/CFP10 and PPD, as well as influenza HA and tetanus titers of various isotypes/subclasses, compared with ATB/HIV− subjects (Fig. 3; Fig. S1 and S2), suggesting a dysfunction in the overall humoral immune response in the setting of active disease.

Importantly, the humoral immune defects observed in the ATB/HIV+ group may in part be attributable to their low CD4^+^ T cell counts (Table 1). A loss of T cell help may contribute to B cell dysregulation and broadly compromised humoral immune responses (50–52). However, remarkably, only antigen-specific IgM titers had a significant positive correlation with CD4^+^ T cell counts in HIV-infected individuals. Given that IgM is the first subclass selected during the induction of novel immune responses (65), these data suggest that new M. tuberculosis-specific antibody responses may continue to emerge in response to HIV infection in the setting of high CD4^+^ T cell levels, responses that may be lost with progressive HIV infection. Of note, HIV-infected individuals have been reported to exhibit a paucity of IgM+ memory B cells (22, 66, 67); furthermore, IgM+ memory B cell counts were observed to have a significant positive correlation with CD4^+^ T cell counts in a cohort of HIV-infected individuals (67). Thus, low antigen-specific IgM titers in ATB/HIV+ individuals could potentially reflect a particularly severe defect in the IgM+ memory B cell compartment precipitated by low CD4^+^ T cell counts. Conversely, the lack of a significant positive correlation between IgG responses and CD4^+^ T cell counts in this cohort suggests that alterations in M. tuberculosis-specific IgG responses may be related to additional immunosuppressive changes in B cell responses during HIV/TB coinfection. Nevertheless, further investigation is needed to dissect the biology underlying the relationships, and lack thereof, between antibody titer and CD4^+^ T cell counts.

Furthermore, while the HIV-associated humoral immune defects appear to be pleiotropic—as antibody titers across a range of antigens and pathogens were reduced in ATB/HIV+ individuals—only a few antigens were analyzed in this study. Additional antigen-specific differences in antibody titer, which may vary in magnitude and directionality, are likely associated with HIV infection and were not captured in this study. Moreover, due to sample limitations, only PPD was utilized to profile functional antibody responses. Future work on analogous cohorts should interrogate antibody functionality directed to other antigens and the intact bacterium. Notwithstanding, FcγR binding, which drives antibody effector functions (68), was measured for each M. tuberculosis antigen, highlighting FcγR binding perturbations across antigen specificities (Fig. S3).

While previous studies highlighted the significant resolving power of antibody profiles in discriminating between ATB and LTBI individuals (32, 58), it was uncertain whether HIV infection may affect this biology. Specifically, previous work identified antibody Fc glycosylation as a key driver of separation between purified IgG from ATB and LTBI individuals, as LTBI individuals displayed less IgG fucosylation—associated with increased ADCC activity—coupled with increased anti-inflammatory galactosylation and sialylation (32). Due to sample limitations, here, antibody Fc glycosylation was not measured, and diluted plasma was utilized in the functional and FcγR binding assays instead of purified IgG, which likely drives the differences observed between the two studies. However, HIV-infected individuals display a significantly higher proportion of bulk, agalactosylated and asialylated antibodies than healthy controls (69, 70). Consequently, while HIV-positive individuals likely exhibit an extremely inflamed bulk antibody Fc glycan profile irrespective of TB disease status, it remains unclear whether antigen-specific glycosylation profiles will remain resolving between ATB and LTBI individuals. Future work should examine antigen-specific antibody Fc glycosylation profiles in the setting of HIV/TB coinfection.

However, remarkably, simply collecting isotype, subclass, and functional data to a small array of M. tuberculosis antigens allowed an accurate resolution of LTBI and ATB individuals in both the HIV-negative and HIV-positive setting (Fig. 6), revealing novel biology about the antigen-specific diversity of humoral immune responses present across distinct infection states. In the absence of HIV infection, ATB individuals separate out from LTBI individuals due to their higher M. tuberculosis-specific antibody responses (Fig. 6), which is consistent with a large body of literature demonstrating stronger antibody responses in the setting of ATB. Furthermore, although broad differences in antigen-specific antibody titers between ATB and LTBI individuals were conspicuously absent in the setting of HIV infection, ATB and LTBI individuals could still be accurately distinguished by their M. tuberculosis-specific antibody profiles, with robust M. tuberculosis-specific IgM responses differentiating the LTBI state (Fig. 6). IgM plays a critical role in immunity, driving direct antimicrobial functions including complement activation, opsonophagocytosis, and agglutination (65, 71). Thus, during HIV/TB coinfection, it is possible that higher levels of antigen-specific IgM may contribute to enhanced M. tuberculosis control in LTBI compared with ATB individuals. However, the imbalance in CD4^+^ T cell counts between the ATB/HIV+ and LTBI/HIV+ groups in this study motivates the conduct of additional linked longitudinal and mechanistic studies for further defining the potential role of IgM in antimycobacterial immunity.

Taken together, this work points to several mechanisms by which HIV may drive the dysfunction of the humoral immune response, potentially weakening immunity to M. tuberculosis. Future work should further interrogate the biologic activity and antimicrobial capacity of antibodies derived from these different infection groups. Notably, for a subset of the humoral changes observed in this study—while statistically significant—the magnitude of difference between infection groups was modest. This was particularly true for changes observed in the PPD response. Thus, while ATB/HIV+ individuals broadly demonstrated weaker M. tuberculosis-specific antibody responses, it remains unclear whether these HIV-associated modifications observed in the M. tuberculosis-specific humoral immune response functionally result in reduced humoral immune pressure on M. tuberculosis. Nevertheless, these findings identify novel immunologic challenges associated with HIV/TB coinfection, and additionally provide a tangible basis with which to leverage the key features of humoral immunity identified, to potentially combat M. tuberculosis disease globally via rational diagnostic or therapeutic design.

## MATERIALS AND METHODS

### Study population and sample collection.

Fifteen individuals with active TB (ATB) and 24 individuals with latent tuberculosis infection (LTBI), which were seropositive for human immunodeficiency virus (HIV), as well as 28 individuals with ATB and 25 individuals with LTBI, which were seronegative for HIV, were recruited in Cape Town, South Africa (Table 1). Neither the duration of HIV infection nor the time at which individuals developed ATB is available. Individuals with LTBI were asymptomatic, with no symptoms of active TB disease, had no history of diagnosis or treatment for active TB disease, and had a positive response to ESAT6/CFP10 pooled peptides by IFN-γ production following overnight stimulation of whole blood. All LTBI/HIV+ participants declared that they were not on antiretroviral therapy (ART) at the time of screening, consenting, and enrollment. All individuals with ATB had either positive sputum smear microscopy, a positive culture for Mycobacterium tuberculosis growth, or both. Six of 15 ATB/HIV+ individuals declared that they were on ART at the baseline visit. The remaining ATB/HIV+ individuals declared that they were not on ART at the time of screening, consenting, and enrollment. Blood was obtained from individuals with active TB disease within the first 7 days of starting standard course anti-TB treatment. Peripheral blood from each individual was obtained in sodium heparin Vacutainer tubes (BD Biosciences), and plasma was isolated within 4 h of collection by centrifugation. All participants had given written, informed consent prior to the study, which was approved by the Human Research Ethics Committee of the University of Cape Town and the Western Cape Department of Health and the study institutional review board at Massachusetts General Hospital.

For assay negative-control samples, eight HIV seronegative individuals from Boston, MA, were recruited as donors by the Ragon Institute of MGH, MIT, and Harvard. These individuals were prescreened as having low reactivity to M. tuberculosis antigens, and thus, their raw data were not included in graphical comparisons between TB infection groups. Blood from these individuals was collected in acid citrate dextrose tubes, and plasma was obtained by Ficoll-Histopaque density centrifugation. All donors provided written, informed consent, and the study was approved by the institutional review board at Massachusetts General Hospital.

### Bulk plasma immunoglobulin measurement.

Plasma levels of IgG1, IgG2, IgG3, IgG4, IgA, and IgM were assessed with the Milliplex Map human immunoglobulin isotyping magnetic bead kit (EMD Millipore) according to the manufacturer’s protocol. In short, 50 μl of plasma was diluted 1:16,000 and was added to IgG1, IgG2, IgG3, IgG4, IgA, and IgM detection beads of various fluorescent regions in a clear bottom 96-well plate (Greiner) and incubated for 1 h shaking at room temperature (RT). Following the primary antibody incubation, the beads were washed, and then 25 μl of biotinylated anti-human-κ and anti-human-λ light-chain detection antibody was added and incubated with shaking for 30 min at RT. A total of 25 μl of streptavidin-phycoerythrin (PE) was then added to each well, and the plates were incubated with shaking for 30 min at RT. Following the incubation, the supernatant was removed from each well, and the beads were resuspended in sheath fluid (Luminex Corporation). Finally, PE levels were measured by the Bio-Plex 200 system (FlexMap 3D; Bio-Rad). Samples were measured in duplicate.

### Antigens.

Antigens derived from M. tuberculosis, HIV, and various control pathogens were utilized across multiple assays. An HIV-1 clade B/C consensus gp120 antigen was acquired from Immune Technology. PPD was received from the Statens Serum Institute. Purified LAM, ESAT6, CFP10, Ag85A, and Ag85B were all acquired from BEI Resources. Tetanus toxoid was received from Massachusetts Biologics. PPSV23 is the pneumococcal 23-valent vaccine from Merck Sharp & Dohme Corporation. A pool of recombinant influenza hemagglutinin (HA) antigens (HA1-B/Florida/4/2006, HA-B/Malaysia/2506/2004, H1N1-A/Solomon island/3/2006, H3N2-A/Wisconsin/67/X-161/2005, H3N2-A/Brisbane/10/2007, H1N1-A/New Caledonia/20/99, and H1N1-A/Brisbane/59/2007; Immune Technologies) representing dominant strains from the past 10 years were combined to generate the influenza HA control antigen.

### Antigen-specific immunoglobulin levels.

A custom multiplexed Luminex assay was performed to measure relative antigen-specific immunoglobulin isotype and subclass levels present in the plasma of each individual, as described previously (44) and according to the protocol of the manufacturers (Luminex Corporation), with minor changes. Specifically, magnetic carboxylated fluorescent beads (Luminex Corporation) were coupled to each protein antigen in a two-step carbodiimide reaction. Beads were washed and then activated by resuspension in activation buffer (100 mM monobasic sodium phosphate [pH 6.2]), as well as 50 mg/ml *N*-hydroxysulfosuccinimide (Sulfo-NHS; Pierce) dissolved in ddH_2_0 and 1-ethyl-3-[3-dimethlyaminopropyl]carbodiimide-HCl (EDC; Pierce) dissolved in activation buffer. This solution was rotated for 30 min at RT. Activated beads were then washed three times in coupling buffer (50 mM morpholineethanesulfonic acid [MES; pH 5.0]), and each protein antigen was individually added to the activated beads. This bead solution was rotated for 2 h at RT. Following bead coupling, the beads were isolated via magnetic separation and blocked by rotating incubation at RT for 30 min in phosphate buffered saline (PBS)-TBN (0.1% bovine serum albumin [BSA], 0.02% Tween 20, and 0.05% azide [pH 7.4]). Finally, the protein-coupled beads were washed to remove PBS-TBN and were resuspended in PBS with 0.05% sodium azide for storage at 4°C. LAM and PPSV23 were COOH-4-(4,6-dimethoxy[1,3,5]triazin-2-yl)-4-methyl-morpholinium (DMTMM)-coupled to Luminex microspheres using a protocol described previously (72). In brief, 10 μl of DMTMM (200 mg/ml; Sigma-Aldrich) was added to 125 μg of LAM, and 12 μl of DMTMM was added to 150 μg of PPSV23. These solutions were incubated for 1 h at RT. Excess DMTMM was then removed via Sephadex G-25 PD-10 desalting columns (GE Healthcare) according the instructions of the manufacturer. Then, 125 μg of DMTMM-activated LAM or 150 μg of DMTMM-activated PPSV23 was each added to 9 million Luminex microspheres.

To perform the Luminex assay using the antigen-coupled beads, each bead was mixed to a concentration of 100 beads/μl per antigen in 0.1% BSA-PBS, and 2,500 beads per antigen per well were added to a clear, flat-bottom 384-well plate (Greiner). A total of 50 μl plasma (1:100 dilution in PBS for IgG1 to 4 and 1:50 dilution for IgA1 to 2 and IgM) was added to the wells and incubated shaking at 800 rpm overnight at 4°C. The plate was then washed 6 times with assay buffer, and 40 μl of PE-conjugated mouse anti-human total IgG, IgG1, IgG2, IgG3, IgG4, IgA1, IgA2, or IgM (Southern Biotech) at 1.3 μg/ml was added and incubated with shaking at 800 rpm at RT for 1 h. The plate was then washed 6 times with sheath fluid (Luminex Corporation) and resuspended in sheath fluid in a final volume of 60 μl. PE median fluorescence intensity (MFI) levels were then measured via the iQue screener plus (Intellicyt) system and analyzed by Forecyt standard edition version 6.1.6465. Samples were measured in duplicate.

### Antigen-specific Fcγ receptor binding.

Recombinant Fcγ receptors (FcγRs) were used to determine the relative binding levels of antigen-specific antibodies to various FcγRs, as described previously (73). In brief, Avi-tagged FcγRIIa(H), FcγRIIIa(V), and FcγRIIb from the Duke Human Vaccine Institute were biotinylated with a BirA biotin-protein ligase standard reaction kit (BirA500; Avidity) according to the manufacturers protocol, and excess biotin was removed by Zeba spin desalting columns (7K MWCO; Thermo Fisher Scientific). Antigen-coupled Luminex microspheres were then added to the 1:100-diluted plasma samples, incubated overnight at 4°C shaking, and then washed 6 times in assay buffer as described above. Prior to the addition of each FcγR to the immune-complexed microspheres, streptavidin-R-phycoerythrin (ProZyme) was added to each FcγR in a 4:1 molar ratio and incubated for 20 minutes at RT to allow fluorescent labeling of the FcγRs. Immediately after, 40 μl of the FcγRs (1 μg/ml in 0.1% BSA-PBS) was added to the immune-complexed microspheres. The FcγRs were incubated with the microspheres for 1 h at RT and washed 6 times, and then the median PE intensity was measured via the iQue screener plus (Intellicyt) and analyzed by Forecyt standard edition version 6.1.6465. Samples were measured in duplicate.

### Antibody-dependent cellular phagocytosis.

The ability of plasma from each individual to drive phagocytosis of PPD-coupled beads by THP-1 monocyte cells was determined as described previously (32, 74). In brief, for every 100 μg of PPD, 26.6 μl of 10 mM EZ-Link sulfo-NHS-LC-biotin (Thermo Fisher Scientific) was added to biotinylate the antigen. After a 30-minute incubation at RT, excess biotin was removed using Amicon Ultra 0.5-ml columns (3K; Millipore Sigma). Biotinylated PPD was then added to fluorescein isothiocyanate (FITC)-conjugated neutravidin beads (1.0 μm, Invitrogen) at a ratio of 1 μg antigen:1 μl beads and incubated for 2 h at 37°C. Following the incubation, unbound antigen was washed away, and 10 μl of plasma diluted to 1:30 was added to 10 μl of antigen-coupled neutravidin beads and incubated for 2 h at 37°C. The beads were washed, and 200 μl THP-1 cells (2.5 × 10^4^ cells per well) were added to the beads and incubated for approximately 18 h at 37°C. Following the incubation, THP-1 cells were fixed in 4% paraformaldehyde (PFA), and fluorescent bead uptake was measured on the iQue screener plus (Intellicyt). Data were analyzed in FlowJo 10.3. Phagocytic scores were calculated as [(%FITC-positive cells) × (geometric mean fluorescence intensity of the FITC positive cells)] divided by 10,000. Samples were run in duplicate.

### Antibody dependent neutrophil phagocytosis.

The ability of plasma from each individual to drive phagocytosis of PPD-coupled beads by primary human neutrophils was determined as described previously (75). In brief, PPD was biotinylated and coupled to fluorescent neutravidin beads (1.0 μm; Invitrogen), incubated with plasma, and washed as described above for ADCP. During the 2-hour bead and plasma incubation, fresh peripheral blood collected from healthy donors in acid citrate dextrose anticoagulant tubes was added at a 1:9 ratio to ACK lysis buffer (150 mM NH_4_Cl, 8,610 mM KHCO_3_, and 0.1 mM Na_2_-EDTA [pH 7.4]) for 5 minutes at RT. After red blood cell lysis, the blood was centrifuged for 5 minutes at 1,500 rpm. After centrifugation, the supernatant was removed, and leukocytes were washed with 50 ml of 4°C PBS, spun for 5 minutes at 1,500 rpm, and resuspended in R10 medium (RPMI [Sigma], 10% fetal bovine serum [Sigma], 10 mM HEPES [Corning], and 2 mM l-glutamine [Corning]) at final concentration of 2.5 × 10^5^ cells/ml. Leukocytes (5 × 10^4^ cells/well) were then added to the immune-complexed beads and incubated for 1 h at 37°C and 5% CO_2_. Following this incubation, the plates were spun for 5 minutes at 500 g to pellet the cells. After removal of the supernatant, anti-human CD66b-Pacific Blue (Biolegend) was added to the leukocytes, and the cells were incubated for 20 minutes at RT. Following this incubation, the cells were washed with PBS and fixed with 4% PFA. Finally, fluorescent bead uptake was measured on the iQue screener plus (Intellicyt) in the CD66b-positive cell population, and data were analyzed in FlowJo 10.3. Phagocytic scores were calculated as described above. ADNP assays were performed with blood from two different donors.

### Antibody-dependent natural killer cell activation.

An enzyme-linked immunosorbent assay (ELISA)-like assay was performed to measure NK cell degranulation via CD107a expression (76) and NK cell activation via intracellular production of interferon gamma (IFN-γ) and macrophage inflammatory protein 1 beta (MIP1β), as described previously (32). In short, ELISA plates (Thermo Fisher Nunc MaxiSorp flat bottom) were coated with 300 ng/well of PPD and incubated overnight at 4°C. The plates were then washed with PBS and blocked with 5% BSA-PBS for 2 h. Next, the plates were again washed with PBS, and 50 μl of 1:30 diluted plasma was added to incubate for 2 h at 37°C. One day prior to adding the diluted plasma, NK cells were isolated from the whole blood of healthy donors using the RosetteSep human NK cell enrichment cocktail (Stemcell Technologies) and Sepmate conical tubes (Stemcell Technologies) according to the instructions of the manufacturer. Following isolation, NK cells were incubated overnight at 1.5 × 10^6^ cells/ml in R10 medium with 1 ng/ml interleukin-15 (IL-15). After the 2-h plasma incubation, the assay plates were washed, and 50,000 primary human NK cells, together with 2.5 μl PE-Cy5 anti-human CD107a (BD), 0.4 μl Brefeldin A (5 mg/ml; Sigma), and 10 μl GolgiStop (BD), were added to each well of the assay plates. The plates were then incubated for 5 h at 37°C. Following the incubation, the samples were stained with 1 μl each of PE-Cy7 anti-human CD56, APC-Cy7 anti-human CD16, and Alexa Fluor 700 anti-human CD3 (all from BD). After a 20-minute incubation at RT to allow extracellular staining, the plate was washed with PBS and the cells were fixed using Perm A and Perm B (Invitrogen). The Perm B solution additionally contained PE anti-human MIP-1β and APC anti-human IFN-γ (both from BD) to allow intracellular cytokine staining. After a final wash in PBS, the cells were resuspended in PBS, and the fluorescence of each marker was measured on a BD LSR II flow cytometer (BD Biosciences) and analyzed by FlowJo 10.3. NK cells were defined as CD3-negative, CD16-positive, and CD56-positive cells. The assay was performed using NK cells from 3 different donors.

### Statistics.

Kruskal-Wallis with Dunn’s multiple-comparison test was utilized throughout univariate analyses for adjusted *P* value calculations. These statistics were performed in GraphPad Prism 8.4.0.

The Benjamini-Hochberg procedure was utilized to calculate *q* values for the correlation analyses and the ATB/HIV+ analysis stratified by ART status (77). These calculations were performed in R (version 3.6.2).

### Classification of infection states.

Classification models were generated to determine whether individuals with different TB clinical states (ATB and LTBI) could be distinguished in the presence/absence of HIV coinfection, using antibody profiles alone. Least absolute shrinkage selection operator (LASSO) (56) was initially utilized for M. tuberculosis-specific antibody feature selection, followed by PLS-DA classification using the LASSO-selected features (57, 78).

For feature selection, 100 stratified random samples each comprising of 90% of the individuals were generated. A LASSO model, in which the penalty term lambda was chosen via 5-fold cross-validation, was then fit on each random sample. Features selected in at least 75% of the LASSO models were moved forward to use for classification. Statistical significance of the features is the univariate significance determined by Kruskal-Wallis with Dunn’s multiple-comparison test.

PLS-DA models were then fit using the LASSO-selected features, and the model accuracy ([1 – balanced error rate] × 100) calculated during 5-fold repeated cross-validation was used to evaluate model performance and is reported. The significance of each model was additionally assessed via permutation testing. Specifically, first, the group labels of the individuals were randomly permuted. Following group label permutation, a PLS-DA model was fit and evaluated for model accuracy in a 5-fold cross-validation framework. This process was repeated 100 times, and then the model accuracy of the real model was compared with that of the permuted models with a Mann-Whitney U test to assess model significance.

Visualizations of latent variables from the PLS-DA model are included, as is variable importance in the projection plots, indicating the relative contribution of individual features to the first latent variable. LASSO was implemented using the glmnet package (version 3.0-2) in R (version 3.6.2) (79). PLS-DA models were implemented using the mixOmics package (version 6.10.9) in R (version 3.6.2) (78).

### Correlation networks.

Data were separated into two groups, namely, HIV-positive and HIV-negative individuals. Next, Spearman correlations between each M. tuberculosis-specific antibody feature measured were performed. Significant correlations between antibody features (*q* value of <0.01) were then used to populate a correlation network in which the thickness of the edges corresponds to the magnitude of the Spearman *r* value for the given relationship. Spearman correlations and *q* values were computed in R (version 3.6.2). Correlation matrices were generated in Cytoscape.

### Data availability.

Any materials, data, and R code will be made available to members of the scientific community in a timely fashion following a reasonable request. We guarantee our authority to comply with this policy.
