# Supplementary figures and images for: HIV Is Associated with Modified Humoral Immune Responses in the Setting of HIV/TB Coinfection
Source: mSphere. 2020 May 20;5(3):e00104-20. doi: 10.1128/mSphere.00104-20 (PMC7380575; doi:10.1128/mSphere.00104-20)

A

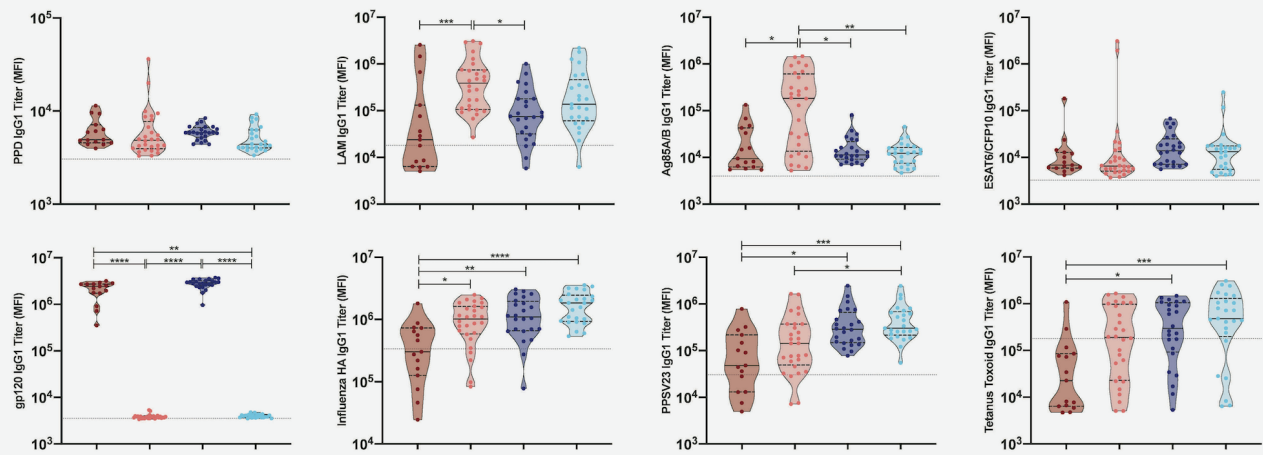

B

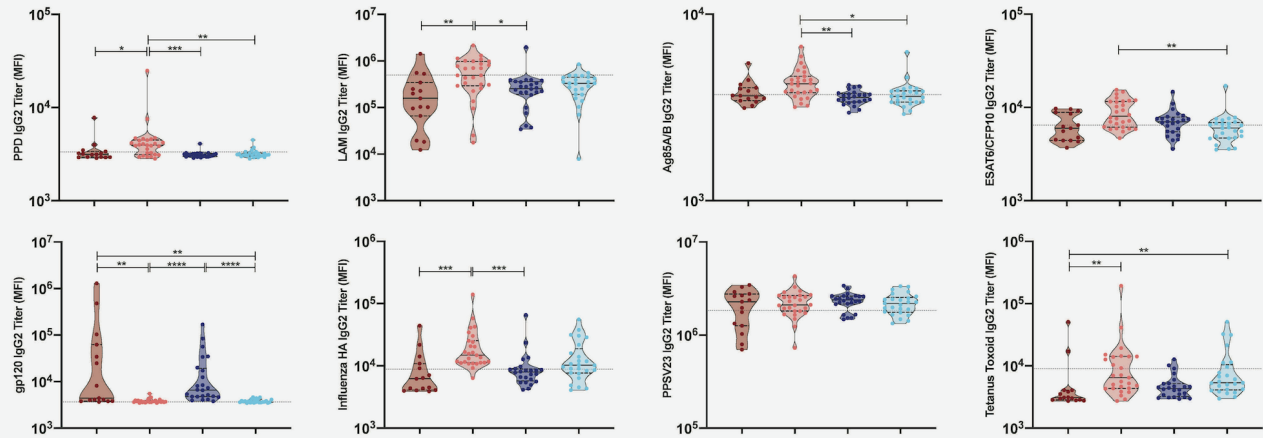

C

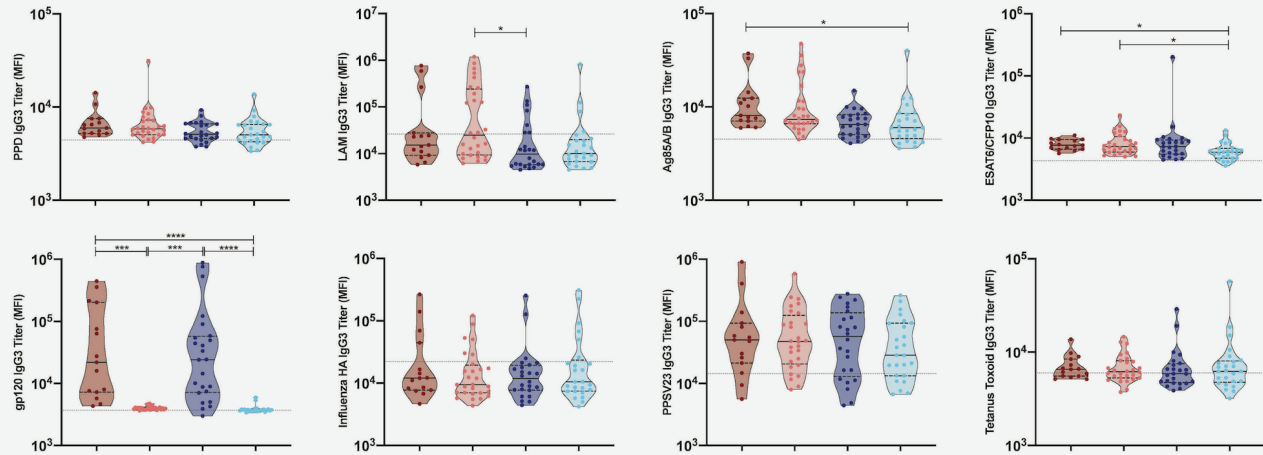

D

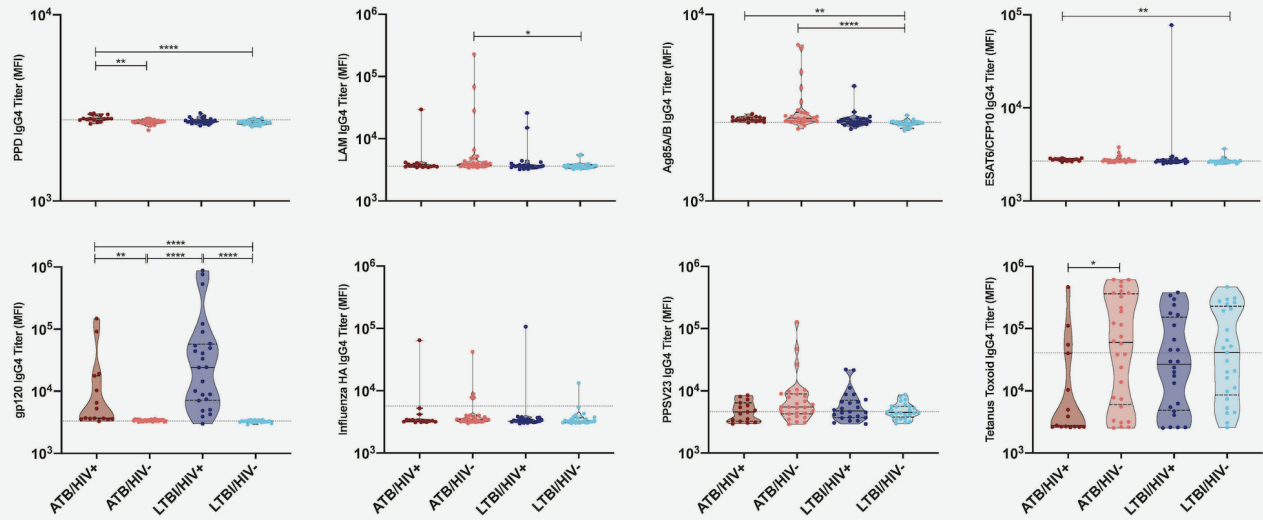

Supplement: FIG S1 [file mSphere.00104-20-sf001.pdf]

A

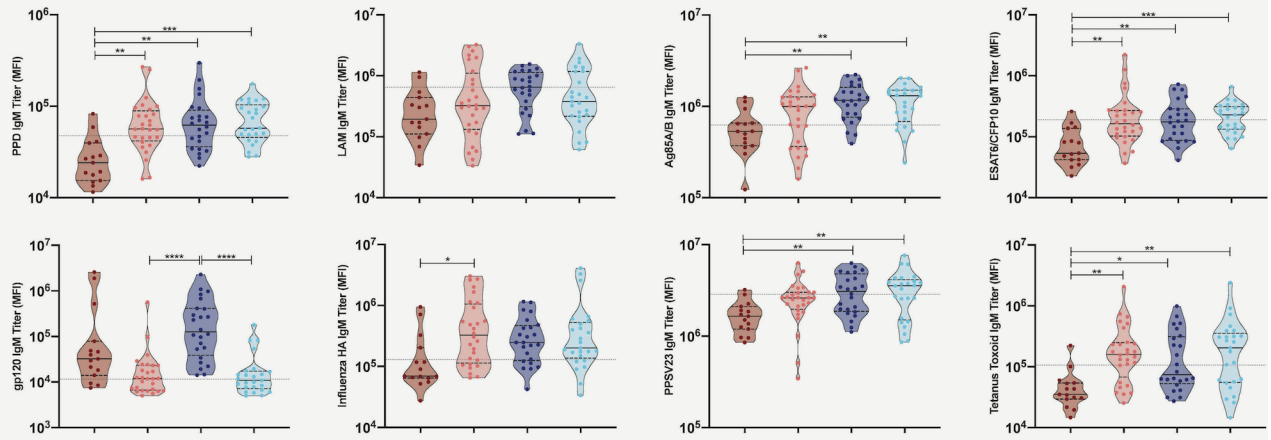

B

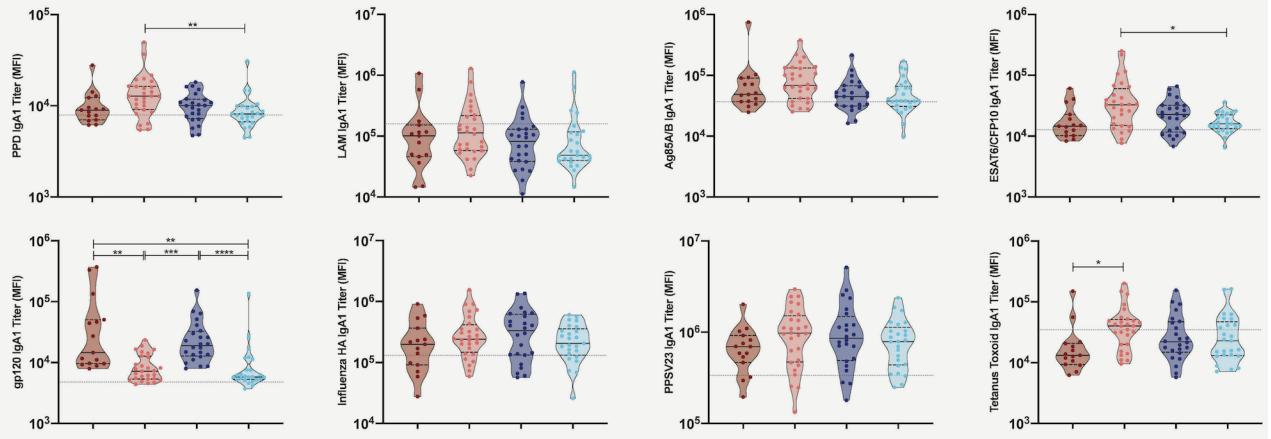

C

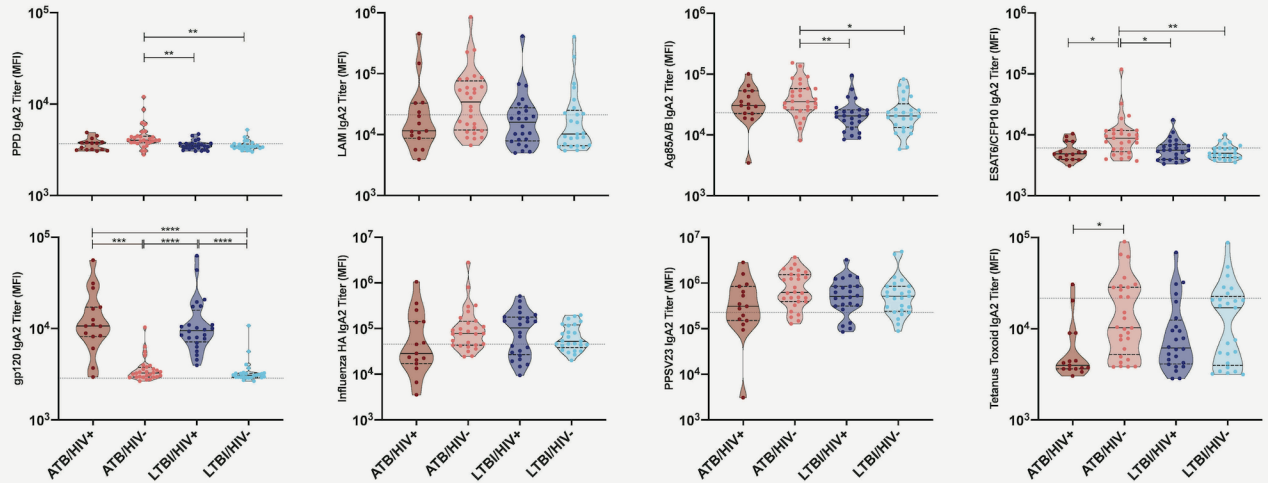

Supplement: FIG S2 [file mSphere.00104-20-sf002.pdf]

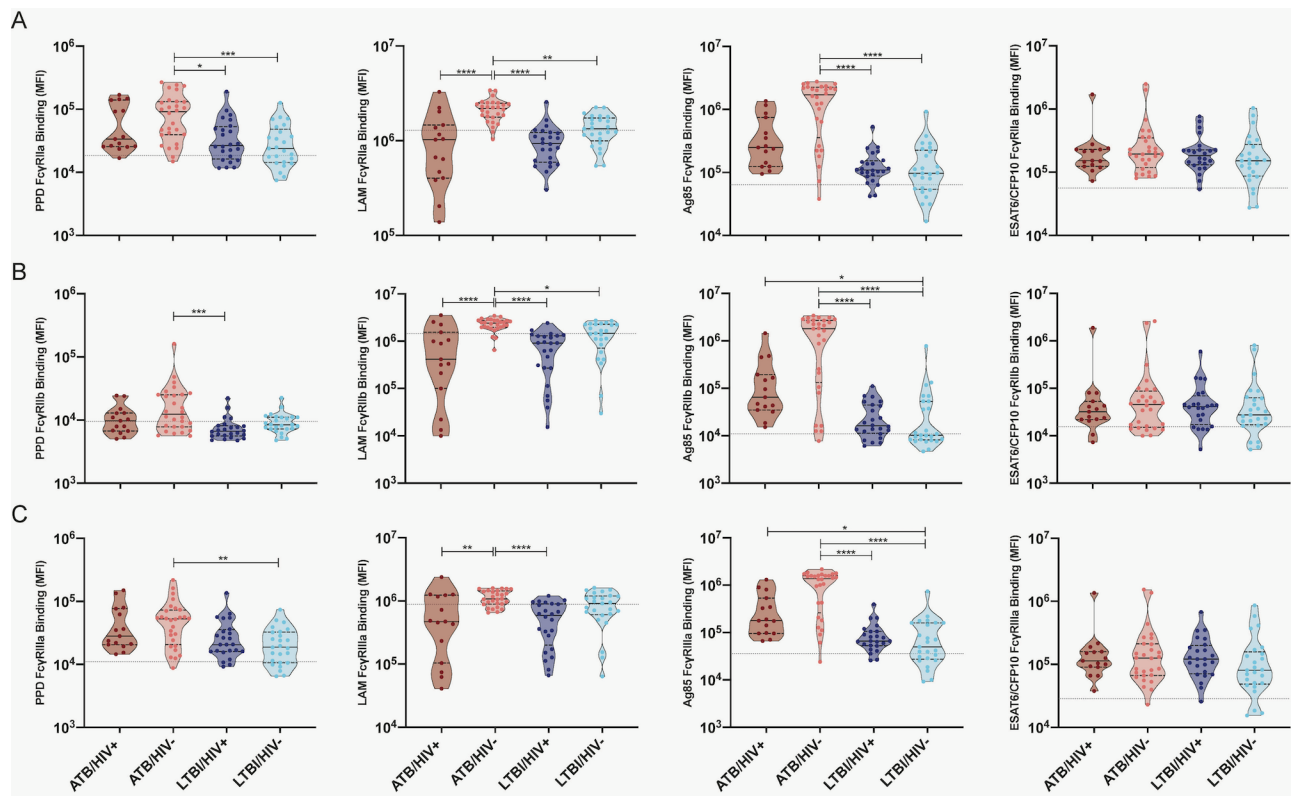

Supplement: FIG S3 [file mSphere.00104-20-sf003.pdf]

A

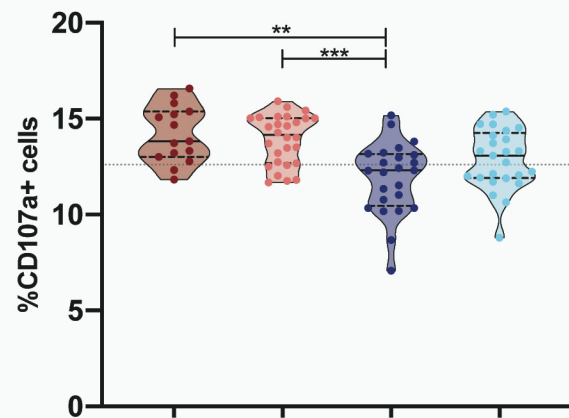

B

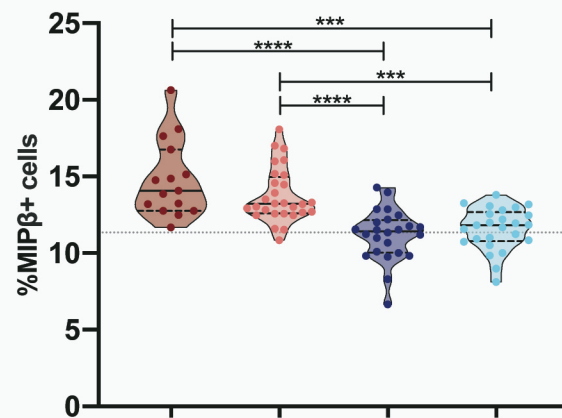

C

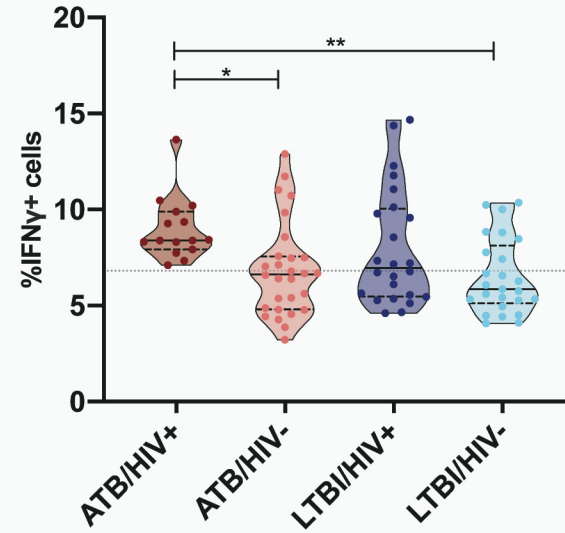

D

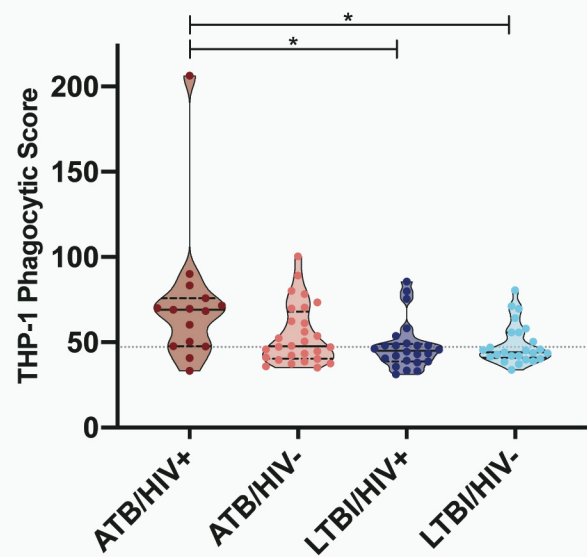

E

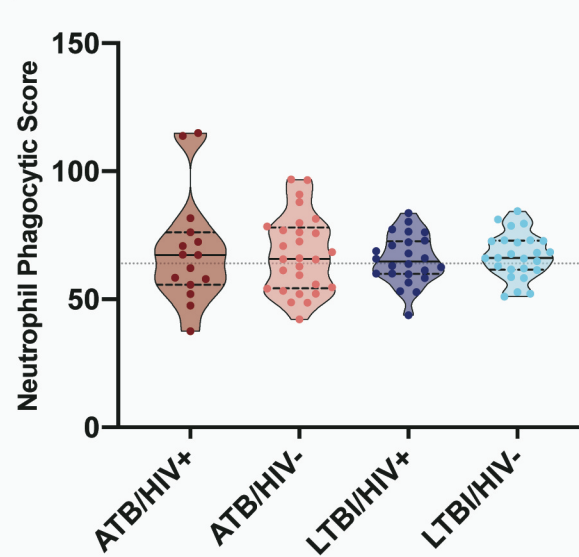

Supplement: FIG S4 [file mSphere.00104-20-sf004.pdf]
