# Supplementary material for: HIV Is Associated with Modified Humoral Immune Responses in the Setting of HIV/TB Coinfection
Source: mSphere. 2020 May 20;5(3):e00104-20. doi: 10.1128/mSphere.00104-20 (PMC7380575; doi:10.1128/mSphere.00104-20)
Supplement: TABLE S1 [file mSphere.00104-20-st001.pdf]

Feature	p-value	q-value
viral_load	0.0294	0.8873
ESAT6.CFP10.IgG4	0.0677	0.8873
LAM.total.IgG	0.1116	0.8873
PPD.IgA1	0.2159	0.8873
ESAT6.CFP10.IgM	0.2159	0.8873
Ag85.IgM	0.2629	0.8873
PPD.IgG2	0.3165	0.8873
PPD.IgA2	0.3165	0.8873
ESAT6.CFP10.IgA1	0.3768	0.8873
LAM.IgG3	0.3768	0.8873
CD4_count	0.3785	0.8873
PPD.IgG4	0.4437	0.8873
PPD.IgM	0.4437	0.8873
Ag85.IgG2	0.4437	0.8873
Ag85.IgG4	0.4437	0.8873
Ag85.IgA1	0.4437	0.8873
LAM.IgG1	0.4437	0.8873
ESAT6.CFP10.IgA2	0.5169	0.9763
Ag85.total.IgG	0.5959	1.0000
LAM.IgG2	0.5959	1.0000
Ag85.IgG3	0.6800	1.0000
LAM.IgA2	0.6800	1.0000
ESAT6.CFP10.IgG1	0.7683	1.0000
PPD.IgG1	0.8597	1.0000
Ag85.IgA2	0.8597	1.0000
ESAT6.CFP10.IgG3	0.8597	1.0000
PPD.total.IgG	0.9530	1.0000
PPD.IgG3	0.9530	1.0000
Ag85.IgG1	0.9530	1.0000
ESAT6.CFP10.IgG2	0.9530	1.0000
LAM.IgM	0.9530	1.0000
ESAT6.CFP10.total.IgG	1.0000	1.0000
LAM.IgG4	1.0000	1.0000
LAM.IgA1	1.0000	1.0000
